# The astrocyte transcriptome in EAE optic neuritis shows complement activation and reveals a sex difference in astrocytic C3 expression

**DOI:** 10.1038/s41598-019-46232-6

**Published:** 2019-07-10

**Authors:** Alessia Tassoni, Vista Farkhondeh, Yuichiro Itoh, Noriko Itoh, Michael V. Sofroniew, Rhonda R. Voskuhl

**Affiliations:** 1 Multiple Sclerosis Program, Department of Neurology, David Geffen School of Medicine, University of California, Los Angeles, CA 90095 USA; 2 Department of Neurobiology, University of California, Los Angeles, CA 90095 USA

**Keywords:** Neurology, Neuroscience

## Abstract

Multiple sclerosis (MS) is a neuroinflammatory multifocal disorder. Optic neuritis is common in MS and leads to visual disability. No current treatments repair this damage. Discerning gene expression changes within specific cell types in optic nerve (ON) may suggest new treatment targets for visual disability in MS. Astrocytes are pivotal regulators of neuroinflammation, playing either detrimental or beneficial roles. Here, we used RiboTag technology to characterize the astrocyte-specific transcriptome in ON in the experimental autoimmune encephalomyelitis (EAE) model of MS. RNA sequencing analysis showed the Complement Cascade and Cholesterol Biosynthesis Pathways as the most enriched and de-enriched pathways, respectively, in ON astrocytes in EAE. Expression of complement component 3 (C3) was confirmed to be increased in ON astrocytes at the protein level during EAE. A bigger increase in C3 expressing ON astrocytes was found in EAE females versus healthy females, as compared to that in EAE males versus healthy males. Also, there was worse retinal ganglion cell (RGC) and axonal loss in EAE females. Regression analyses showed a negative correlation between C3 expressing astrocytes and RGC density. This cell-specific and sex-specific investigation of the optic nerve provides targets for the development of therapeutic strategies tailored for optic neuritis in MS.

## Introduction

The central nervous system (CNS) contains a variety of different cell types, whose composition and gene expression are altered during disease. Multiple sclerosis (MS) is characterized by immune cell infiltration, glia reactivity, demyelination, axonal damage and synaptic loss. MS patients experience a variety of disabilities in vision, walking, coordination, and cognition, with heterogeneity between patients regarding which disabilities are most severe, particularly early in disease. The neurodegenerative aspect of each disability likely has distinct features, since molecules and cells within each neurological pathway are not identical. Thus, we previously hypothesized that each neurological pathway needs to be specifically investigated in order to identify treatments tailored for each disability^1^. Using a cell- and region-specific approach^2–4^, we showed regional differences in astrocyte gene expression among CNS regions in the experimental autoimmune encephalomyelitis (EAE) mouse model of MS^1^. We found that cholesterol synthesis pathways were significantly downregulated in white matter rich region astrocytes (spinal cord, cerebellum and optic nerve) in EAE. Since cholesterol in astrocytes is transported via ApoE to oligodendrocytes to make myelin, and to neurons to make membranes and synapses, we treated EAE mice to reverse this abnormality. Treatment with an agonist for ATP-binding cassette transporter A1 (ABCA1) increased cholesterol synthesis in spinal cord astrocytes and improved motor function as shown using standard EAE walking scores and rotarod performance. Here, we extend our cell-specific and region-specific approach to optic nerve (ON), since optic neuritis is frequent in MS^5^.

Current MS treatments are anti-inflammatory and reduce relapses, thereby providing indirect neuroprotection. However treatments targeting CNS cells are needed for direct neuroprotection^6^. Visual disability in MS accumulates following recurrent attacks of optic neuritis. This entails inflammation and demyelination of the ON, axonal damage and loss, and retinal ganglion cell (RGC) death. Optic neuritis in EAE has been used as a model of optic neuritis in MS^7,8^. Previous studies suggested astrocytes as key players in orchestrating visual disability progression in EAE^9,10^. Astrocytes are heterogeneous across the CNS and are pivotal regulators of neuroinflammation, neurodegeneration and repair in CNS disorders^1,11–13^. Depending on severity, timing and context of the injury, astrocytes undergo molecular and functional changes and can play both beneficial and detrimental roles^13–15^. Several studies have shed light on potential mechanisms behind the “Janus face” of astrocytes under several pathological conditions^16–18^; however, roles of astrocytes in ON during optic neuritis remain unclear. Here, we characterized the astrocyte-specific transcriptome in ON in EAE using RiboTag technology. Such technology uses the Cre-LoxP recombination to generate mice expressing hemagglutinin (HA)-tagged ribosomes in specific cell types^1,2,4^. GFAP-Cre RiboTag mice enabled us to isolate astrocyte specific transcripts from ON, overcoming limitations inherent to interpretation of gene expression analysis performed on whole tissues, characterized by variability in cell composition during disease. Our RNA sequencing data revealed the importance of complement component C3 in ON astrocytes. Finally, sex as a biological variable was examined comparing female and male mice with optic neuritis.

## Results

### Optic nerve pathology and astrocyte reactivity in chronic EAE

**Figure 1:**
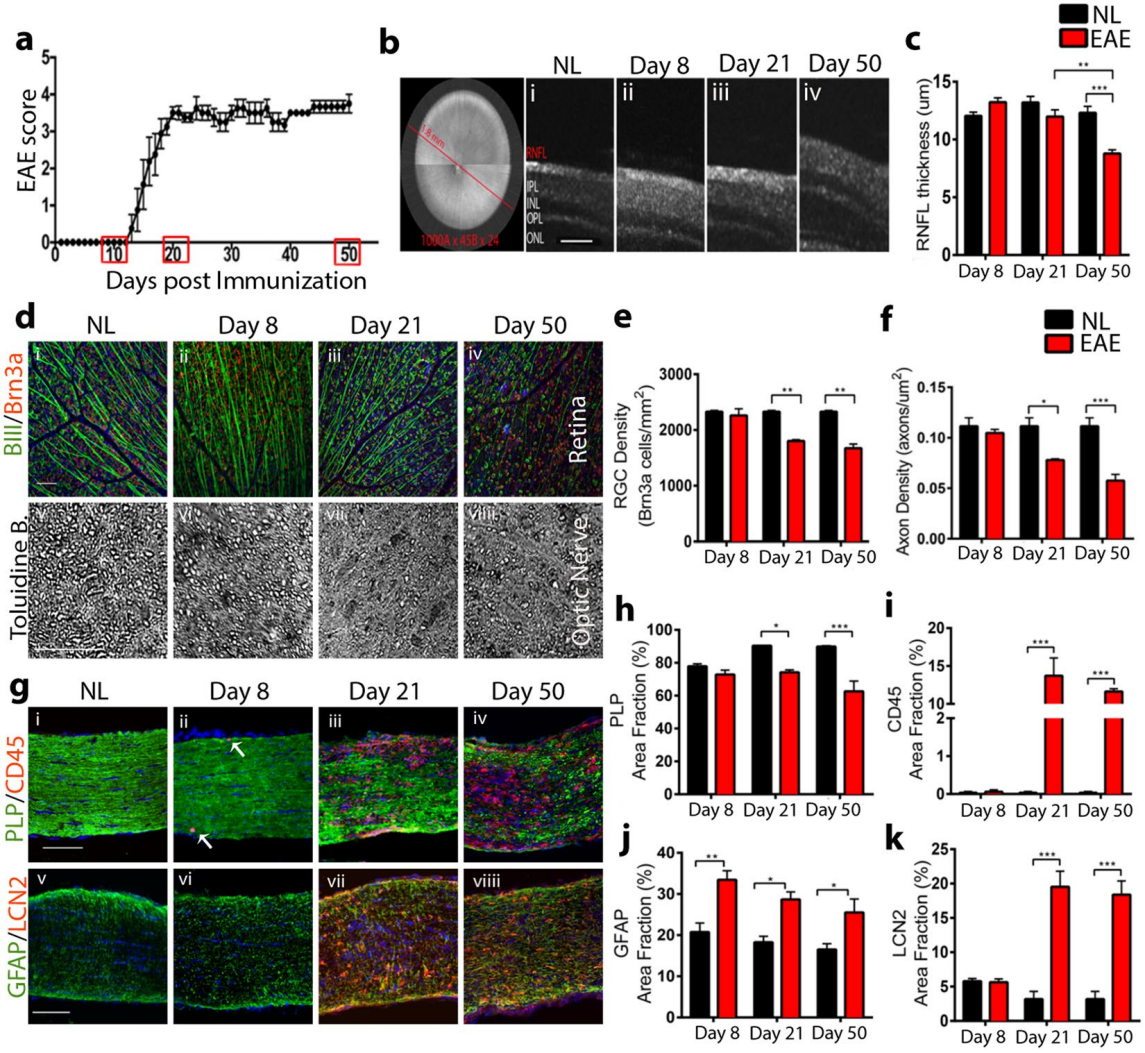
ON pathology and astrocyte reactivity in chronic EAE. (**a**) EAE clinical score. Highlighted in red are the time points investigated (day 8, 21, 50). (**b**) SD-OCT radial scans of the retina showing time course of RNFL thinning at days 8 (***i***), 21 (***ii***) and 50 (***iii***) post immunization. Scale bar 50 μm. (**c**) Quantitative analysis of RNFL thickness showing significant thinning at EAE day 50 (red bar), compared to NL (black bar). (**d**) Brn3a (red)/BIII tubulin (green) double immunostaining showing time course of RGC body density in the retina (***i***-***iv***); Toluidine blue staining of ON semithin sections showing time course of axonal density (***v***-***viii***). Scale bar 50 um. (**e**,**f**) Quantitative analysis of RGC body (**e**) and axonal (**f**) density showing significant cell body and axonal loss at EAE day 21 and 50 (red bars) compared to NL (black bars). (**g**) PLP (green)/CD45(red) double staining showing time course of myelin loss and reactive microglia /blood derived immune cell distribution, respectively, in ON (***i***-***iv***); GFAP (green)/LCN2 (red) double staining showing time course of astrocyte reactivity in ON (***v***-***viii***). Nuclei were counterstained with DAPI (blue). Scale bar 100 μm. (**h**,**i**) Quantitative analysis of PLP (**h**) and CD45 (**i**) stained area fraction, showing significant loss of myelin integrity and increase in microglia reactivity/immune cell infiltration in ON at EAE day 21 and 50 (red bars), compared to NL (black bars). (**j**,**k**) Quantitative analysis of GFAP (**j**) and LCN2 (**k**) stained area fraction, showing significant increase in astrocyte reactivity during EAE (red bars) compared to NL (black bars), with GFAP uregulation already detectable at EAE day 8 (**j**). For all bar graphs, data represent mean ± s.e.m. (n = 5 per group), Two way ANOVA with Sidak’s multiple comparison test, *p < 0.05, **p < 0.01, ***p < 0,0001.

In order to perform astrocyte transcriptomics analysis in the context of optic neuritis, we first characterized ON pathology in the chronic MOG_35–55_ EAE mouse model of MS at three different time points, namely day 8 (before clinical onset), day 21 (peak of disease) and day 50 (chronic phase) (Fig. 1a). Sex- and aged- matched mice were used as healthy controls (Normal, NL). Spectral domain optical coherence tomography (SD-OCT) and neuropathological examinations were used to investigate neurodegeneration and neuroinflammation. Longitudinal SD-OCT examination of the RNFL showed thinning over the course of the disease, with a significant decrease in RNFL thickness at day 50 in EAE compared to NL mice (Fig. 1b,c). Significant RGC cell body and axonal loss were observed by standard neuropathology in whole mount retina and ON sections, respectively, at EAE days 21 and 50 compared to NL mice (Fig. 1d–f). These data corroborated our OCT results of RNFL thinning at day 50, but also showed that neuropathology is more extensive than suggested by OCT at the earlier day 21 time point. Next, neuroinflammation and demyelination were assessed in EAE and NL mice by double immunostaining of ON sagittal sections for CD45, a marker for microglia and blood-derived inflammatory cells, and proteolipid protein (PLP), a marker for myelin (Fig. 1g_i–iv_). A significant decrease in myelin integrity was observed at EAE days 21 and 50 (Fig. 1g,h) compared to NL, in parallel with a significant increase in blood-derived inflammatory cell infiltration (Fig. 1g,i). This was accompanied by a significant increase in microglia/macrophage reactivity at EAE days 21 and 50, as assessed by immunostaining for MHCII and Iba1 (Supplemental Fig. 4b). Having characterized neurodegeneration and inflammation in ON in EAE, we next determined astrocyte reactivity in this context. We double immune-labelled ON sagittal sections from EAE and NL mice at the defined time points for the astrocyte reactivity marker, glial fibrillary acid protein (GFAP), alongside Lipocalin 2 (LCN2), an autocrine mediator of reactive gliosis thought to contribute to disease progression during EAE^10,19,20^. As shown in Fig. 1g_v–viii_, ON astrocytes upregulated GFAP, indicative of increased reactivity, as early as EAE day 8, when only few blood derived immune cells had infiltrated the ON parenchyma (Fig. 1g_ii_), and its expression was maintained over the course of the disease (Fig. 1g,j). LCN2 expression was highly induced at EAE days 21 and 50, in parallel with other inflammatory and degenerative markers (Fig. 1g,k). Together, these data mapped the reactive astrocyte phenotype relative to inflammation and neurodegeneration in ON during EAE to provide a framework for interpretation of findings from RNA sequencing of ON astrocytes during optic neuritis.

### RNA sequencing profile of optic nerve astrocytes during optic neuritis

**Figure 2:**
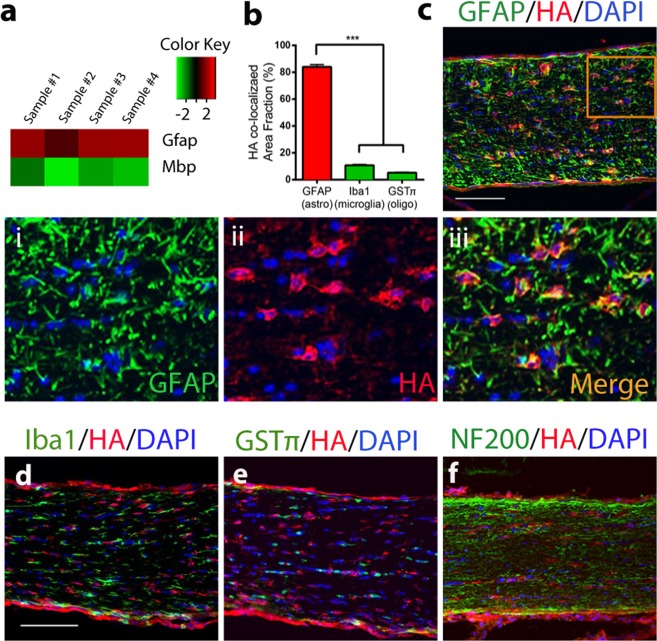
Enrichment of astrocyte-specific mRNA using RiboTag technology. (**a**) Heat map showing enrichment of astrocytic transcript *Gfap* (red) and de-enrichment of oligodendrocyte specific transcript (*Mbp*, green) in HA-IP RNA. Refer to our previously published paper^1^ for further validation by qPCR. (**b**) HA co-localization efficiency in astrocytes (red bar) was shown to be over 80% and significantly higher compared to microglia and oligodendrocytes (Iba1 and GSTπ, respectively, green bars). Data represent mean ± s.e.m. (n = 5 per group), one way ANOVA with Dunnett’s multiple comparison test, ***p < 0.001. (**c**) Immunofluorescence showing co-localization of HA-tag (red) with astrocyte marker GFAP (green) in ON. (***i***–***iii***) Magnification of inset in (**c**), showing (*i*) GFAP (green), (*ii*) HA (red) and (*iii*) merged image (yellow). (**d**–**f**) Immunofluorescence showing non-co-localization of HA-tag (red) with (**d**) microglia (Iba, green), (**e**) oligo (GSTπ, green) and (**f**) axons (NF200, green). Nuclei were counterstained with DAPI (blue). Scale bar 100 μm.

We investigated astrocyte specific transcriptome changes in optic neuritis using RiboTag technology as described^1^, here focusing on ON. By crossing RiboTag mice, which have the ribosomal protein L22 (*Rpl22*) gene flanked by LoxP recombination sites followed by three HA coding sequences, together with mice having Cre recombinase expression driven by the astrocyte specific GFAP promoter (GFAP Cre 73.12 mouse line), we obtained mice having HA tagged ribosomes specifically in astrocytes (GFAP-Cre RiboTag mice)^1^. Specificity of HA targeting to astrocytes in ON was confirmed previously at the RNA and protein levels^1^, and here enrichment of the astrocyte transcript (*Gfap*, red) and de-enrichment of the mature oligodendrocyte marker (*Mbp*, green) was shown in HA-immunoprecipitated RNA (HA-IP RNA) versus flow-through RNA (FT-RNA) in ON, see heat map (Fig. 2a). Efficiency of HA colocalization to astrocytes in ON was over 84%, as determined by quantifying the percentage of HA labelled area colocalizing with GFAP, in comparison to HA colocalization with Iba1 (microglia) and GSTπ (oligodendrocytes) (Fig. 2b). Double immunostaining of ON sagittal sections for HA alongside cell specific markers, namely GFAP (astrocytes), Iba1 (microglia), GSTπ (oligendroglia) and NF200 (axons), confirmed colocalization of HA with GFAP (Fig. 2c_i–iii_), but not Iba1, GSTπ and NF200 (Fig. 2d–f).

**Figure 3:**
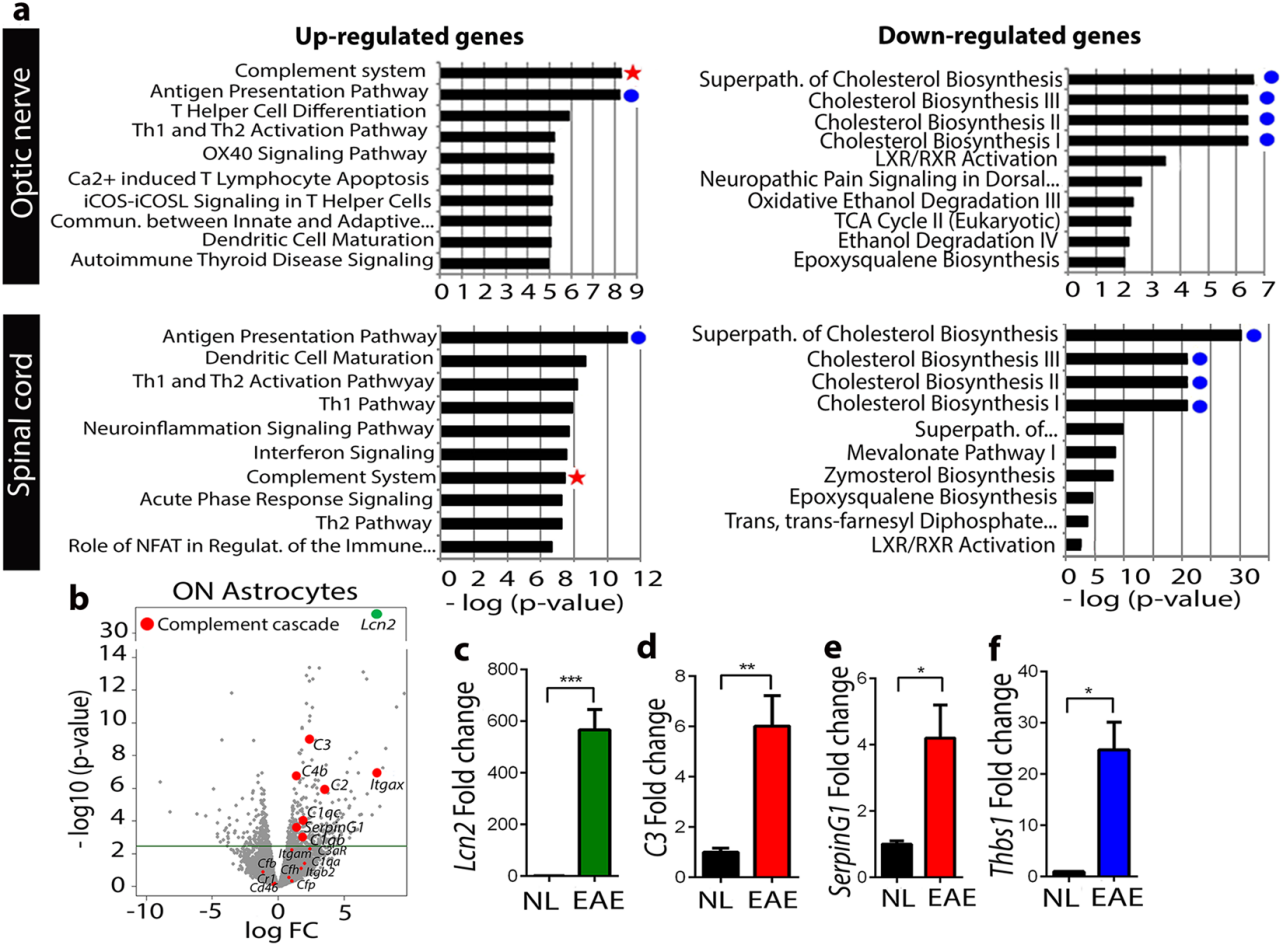
High throughput sequencing and analysis of astrocyte specific RNAs identified the Complement Cascade Pathway as the most significantly uregulated pathway in astrocytes in optic nerve during EAE. (**a**) Pathway analysis showing top 10 most enriched and de-enriched pathways in ON and spinal cord astrocytes of EAE female mice at EAE day 50, compared to NL (n = 4 per group). The Complement Cascade Pathway (red star) was the most significantly upregulated pathway in ON, but not in spinal cord. Cholesterol bioshynthesis pathways (blue dots) were the most significantly downregulated pathways in both spinal cord and ON. (**b**) Volcano plot showing expression of the complement cascade genes (red dots) and the astrocyte reactivity marker *Lcn2* (green dot)) in ON astrocytes at EAE day 50. Complement component *C3* was the most significantly upregulated gene among the complement cascade genes, with a −log10 (p-value) of 9.27; *Lcn2* (green) was the most significantly up-regulated among all genes. (**c**–**f**) Validation of RNA sequencing data by qPCR conducted on a separate set of GFAP-Cre RiboTag mice and confirming significant increase in gene expression level of the rectivity marker *Lcn2* (*c*, green bar), complement cascade genes *C3* and *SerpinG1* (d and e, red bars), and *Thbs1* (f, blue bar) in EAE compared to NL (black bars). Data represent mean ± s.e.m. (n = 5 per group), Student’s t-test, ***p < 0.05, ***< 0.01, ***< 0.0001.

Chronic EAE was induced in female GFAP-Cre RiboTag mice. Healthy GFAP-Cre RiboTag mice were used as controls (NL). ONs were isolated at EAE day 50 and RNA sequencing of astrocyte enriched mRNA immunoprecipitated via the HA tag was performed. Canonical pathway analysis of differentially expressed astrocyte enriched genes revealed over 500 differentially expressed genes (FDR <0.1). Canonical pathway analysis of RNA Seq data from ON astrocytes was first compared to that from spinal cord astrocytes^1^. Interestingly, the Complement Cascade Pathway was the most significantly enriched pathway in ON astrocytes in EAE versus NL, with less dramatic enrichment in spinal cord (Fig. 3a, red star). Similar to spinal cord astrocytes, the Antigen Presentation Pathway was upregulated and several cholesterol biosynthesis pathways were downregulated in ON astrocytes in EAE versus NL (Fig. 3a, blue dots).

To further investigate the most enriched and upregulated pathway in ON, a volcano plot of RNA seq data was generated for genes within the Complement Cascade Pathway (red dots) as well as the astrocyte reactivity marker *Lcn2* (green dot). Among all genes, *Lcn2* was the most significantly upregulated gene in ON astrocytes in EAE compared to NL. Several complement genes were significantly upregulated in ON astrocytes in EAE. Complement *C3* was one of the most significantly upregulated genes in the complement pathway, with a –log10 (p-value) of 9.27. (Fig. 3b). Next, RNA seq results were validated by qPCR using astrocyte HA-IP RNA from a different cohort of EAE and NL GFAP-Cre RiboTag mice. The reactivity marker *Lcn2* showed a significant increase in EAE compared to NL in ON astrocytes (Fig. 3c). The complement genes *C3* and *SerpinG1* were also significantly increased (Fig. 3d,e). Since astrocytic *C3* has been associated with a neurotoxic phenotype of synapse stripping and aberrant phagocytic activity^16,21–23^, while astrocytic *Thbs1* was associated with neuroprotective synaptic plasticity during disease^17,24,25^, we assessed Thbs1 expression in this context of upregulated C3 expression. *Thbs1* expression was also significantly increased in EAE compared to NL in ON astrocytes (Fig. 3f).

### Expression of C3 and THBS1 in optic nerve and retina astrocytes in optic neuritis

**Figure 4:**
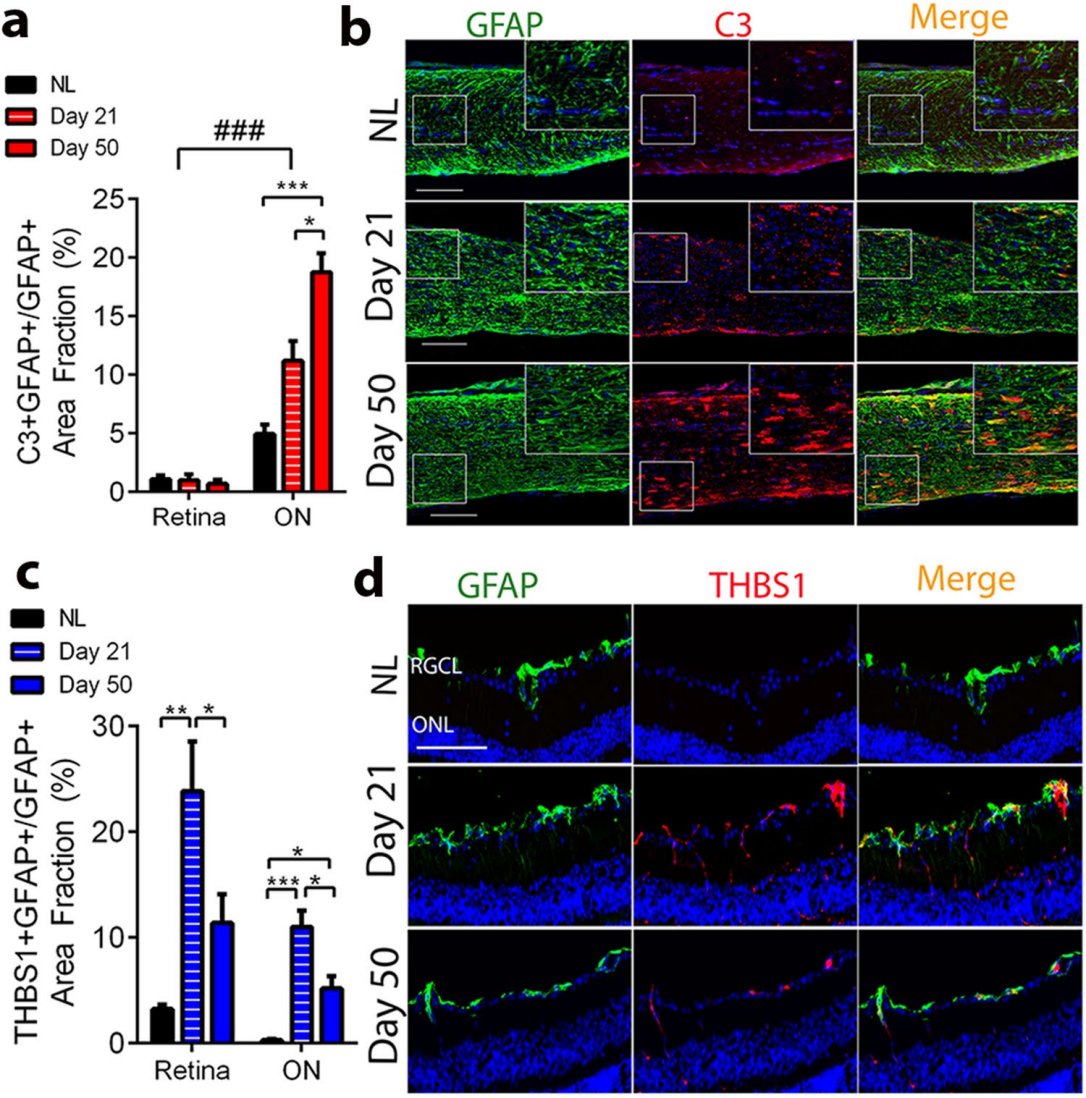
Protein expression of the C3 and THBS1 in ON and retina astrocytes during early and late EAE. (**a**) Quantitative analysis showing significant up regulation of complement component C3 in ON, but not retina, astrocytes at day 21 (red striped bars) and day 50 (red full bars) compared to NL (black bars). (**b**) Immunofluorescence showing expression level of C3 (red) in ON astrocytes (GFAP, green) at EAE day 21 and 50, compared to NL. Nuclei were counterstained with DAPI (blue). Scale bar 100 μm. (**c**) Quantitative analysis showing significant upregulation of THBS1 in both retina and ON astrocytes at day 21 (blue striped bars) compared to NL (black bars), and significant decrease at day 50 (blue full bars) compared to day 21 (blue striped bars). (**d**) Immunofluorescence showing expression level of THBS1 (red) in retinal astrocyte (GFAP, green) at EAE day 21 and 50, compared to NL. Nuclei were counterstained with DAPI (blue). Scale bar 20 μm. For all bar graphs, data represent mean ± s.e.m. (n = 4 per group), One way ANOVA with Tukey’s multiple comparison test, *p < 0.05, **p < 0.01, ***p < 0,0001. Two Way ANOVA with Tukey’s multiple comparison test (Interaction ^#^ < 0.05, ^###^0.001).

Next, we investigated expression of C3 and THBS1 in astrocytes at the protein level in two different regions of the anterior visual pathway, namely ON and retina. The ON, formed by myelinated axonal projections, is where inflammatory lesions and axonal damage occur in EAE optic neuritis. The retina, home of synaptic connections between layers of neurons, lacks myelin and is remote from peripheral immune cell infiltration in ON, but still undergoes axonal and neuronal damage in optic neuritis. Chronic EAE was induced in wild type adult female mice and C3 and THBS1 protein expression was investigated in both regions by immunofluorescence at EAE day 50. Healthy wild type mice were used as controls (NL). Double immunolabelling of sagittal retina and ON sections for C3 with the astrocyte marker GFAP showed increased expression of C3 in EAE ON astrocytes compared to NL (p = 0.0008). In contrast, no increased expression of C3 in astrocytes occurred in retina during EAE compared to NL (Fig. 4a). Representative images of C3 expression in ON and retina are shown in Fig. 4b (GFAP, green; C3, red; Merge, yellow) and Supplemental Fig. 1a, respectively. Further, astrocytic C3 upregulation in ON was accompanied by overexpression of its receptor C3aR in microglia/macrophages, consistent with potential crosstalk between astrocytes and microglia/macrophages during disease^21^ (Supplemental Fig. 2).

We then investigated the astrocytic expression of THBS1 at the protein level. A significant increase in expression of astrocytic THBS1 was observed in retina. This was observed early at EAE day 21 (p = 0.008), but not late at day 50 (p = 0.26), each as compared to NL. Indeed, THBS1 expression decreased from day 21 to day 50 of EAE (p = 0.048). Similar to retina, ON also showed increased astrocytic expression of THBS1 at EAE day 21 (p = 0.0003) and day 50 (p = 0.031) each as compared to NL, with a decrease in its expression from day 21 to day 50 of EAE (p = 0.011) (Fig. 4c). Representative images in Fig. 4d and in Supplemental Fig. 1b demonstrate THBS1 expression in astrocytes in the RNFL and ON (GFAP, green; THBS1, red; Merge, yellow), respectively. To better differentiate astrocytes from Muller cells and define expression of THBS1 in retinal cells, we stained retinal sections for the Muller cell specific marker Retinaldehyde-binding protein 1 (RLBP1), in parallel with GFAP and THBS1. While only astrocytes stain for GFAP in normal retina, activated Muller cells also express GFAP during disease, with GFAP-positive fibers spanning the retina from the inner limiting membrane to the external limiting membrane^26,27^. As shown in Supplemental Fig. 3, astrocytes line the innermost surface of the retina (GFAP^+^ cells), whereas Muller cells (RLBP^+^ cells) span the entire thickness of the retina. In EAE, double positive cells (GFAP^+^ RLBP1^+^) were not found throughout all retinal layers, thereby distinguishing astrocyte staining from Muller cell staining. Thus, the GFAP^+^ cells of the inner retina expressing THBS1 in EAE are in fact astrocytes (Supplemental Fig. 3, arrows).

Together, these data revealed that during optic neuritis, astrocytes in ON undergo important molecular changes, characterized by upregulation of THBS1 in early EAE, and upregulation of C3, in late EAE.

### Sex differences in optic nerve astrocytes during optic neuritis

**Figure 5:**
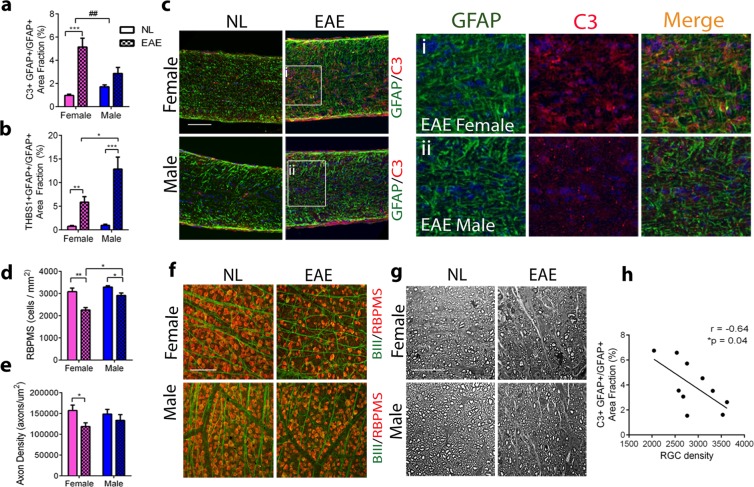
Sex difference in ON astrocytes during optic neuritis. (**a**,**b**), Immunofluorescence quantitative analysis showing sex differences in C3 expressing astrocytes (**a**) and THBS1 expressing astrocytes (**b**) in females (pink bars) and males (blue bars) during EAE (checked bars) compared to NL (full bars). Female astrocytes showed higher expression of C3, while males showed higher expression of the THBS1. (**c**) **i**–**ii**, Representative images showing increased expression of C3 (red) in astrocyte (GFAP, green) in females (i) compared to males (ii) in EAE ON. Nuclei were counterstained with DAPI (blue). Scale bar 100 μm. (**d**) Quantitative analysis of RGC body density showing RGC loss in EAE (checked bars) compared to NL (full bars) in females (pink bars) and males (blue bars), with significantly higher RGC loss occurring in EAE females (checked pink bar) compared to EAE males (checked blue bar). (**e**) Quantitative analysis of axonal loss showing significant loss occurring in females (pink bars) but not in males (blue bars) in EAE (checked bars) compared to NL (full bars). (**f**) Immunofluorescence for RGC markers BIII tubulin (green) and RBPMS (red) showing RGC density in whole mount retina in females and males in EAE and NL. Scale bar 50 μm. (**g**) Toluidine blue staining of ON semithin sections showing axonal density in females and males in EAE and NL. Scale bar 20 μm. (**h**) Significant negative correlation between astrocytic C3 expression and RGC density (Pearson’s correlation coefficient r = −0.64, *p = 0.04). For all bar graphs, data represent mean ± s.e.m. (n = 5–7 per group), Multiple t test *p < 0.05,**p < 0.01, ***p < 0,0001, followed by Two Way ANOVA with Tukey’s multiple comparison test (Interaction ^#^ < 0.05).

Optic neuritis affects more women than men, with a female:male ratio of approximately 3:1 in MS and 7:1 in neuromyleitis optica (NMO)^28,29^. Here, we investigated sex as a biological variable in astrocyte reactivity in optic neuritis in EAE. Chronic EAE was induced in wild type adult females and males. Age-matched healthy wild type females and males were used as controls (NL). Mice were sacrificed at EAE day 50. First, we investigated expression of C3 and THBS1 in astrocytes by immunofluorescence in both sexes. Interestingly, ON astrocytic expression of C3 was significantly increased in EAE females compared to NL females (p = 0.0006), but not in EAE males compared to NL males (p = 0.11), and there was a significant difference in the change with disease within females compared to within males (p = 0.01, Interaction = 0.007) (Fig. 5a). Astrocytic C3 expression is shown in representative images of ON immostained for GFAP (green) and C3 (red) in EAE females, EAE males, and their respective NLs (Fig. 5c_i–ii_). Further, while astrocytic THBS1 expression was significantly increased in ON of both sexes in EAE, the change in THBS1 was significantly higher in EAE males versus NL males than in EAE females versus NL females (p = 0.027, Interaction = 0.06) (Fig. 5b). We then asked whether the sex differences in astrocytic C3 and THBS1 expression was associated with sex differences in ON inflammation and demyelination between sexes. To address this point, we investigated astroglia reactivity, microglia/macrophage reactivity, T cell infiltration and myelin integrity by immunostaining for GFAP, Iba1/MHCII, CD3 and PLP, respectively, in EAE females, EAE males, and their respective age- and sex-matched healthy controls. As expected, a significant increase in the expression of the inflammatory markers and a significant decrease in myelin occured in EAE ONs of both sexes, compared to their respective controls. However, there were no sex differences in these changes (Supplemental Fig. 4a–e). Thus, the sex difference in astrocyte C3 and THBS1 expression did not appear to be driven by a sex difference in ON inflammation and demyelination^16^.

Finally, given the neurotoxic potential of C3^+^ astrocytes and neuroprotective potential of THBS1^+^ astrocytes^14^ we next asked whether the observed sex difference in astrocyte reactive phenotypes was associated with a difference in neurodegeneration in the visual pathway of females and males with optic neuritis. We assessed RGC and axonal loss in whole mount retina and ON sections, respectively, in EAE and NL by immunohistochemistry. Double staining of whole mount retina for RGC marker BIII tubulin (green) and RBPMS (red) showed significant RGC loss occurring in both sexes, but it was worse in females compared to males (p = 0.002, Interaction = 0.08) (Fig. 5d,h). Further, toluidine blue staining of ON semithin sections showed significant axonal loss occurring in EAE females (p = 0.03), but not in males (p = 0.45), when compared to their respective NL controls (Fig. 5e,g). Further, we performed a regression analysis using both male and female mice at EAE day 50 and found a significant negative correlation between astrocytic C3 expression and RGC density (Pearson’s correlation coefficient r = −0.64, *p = 0.04) (Fig. 5h). Finally, we extended our investigation to spinal cord tissue and ran a parallel anaysis of C3 expression in spinal cord white matter astrocytes by immunofluorescence (Supplemental Fig. 5). Astrocytic C3 expression is shown in representative images of spinal cord tissue immostained for GFAP (green) and C3 (red) in EAE females, EAE males, and their respective NLs (Supplemental Fig. 5a_i–ii_). No sex difference in white matter astrocytic C3 expression was observed in spinal cords (Supplemental Fig. 5b), and there was no sex difference in EAE clinical scores, (Supplemental Fig. 5c), consistent with previous reports of no sex difference in EAE scores in C57BL/6 mice^30,31^.

Together, ONs of females as compared to males, had higher percentage of C3 expressing astrocytes and lower percentage of THBS1 expressing astrocytes. This was associated with worse neurodegeneration in the visual pathway of females as compared to males. There was a negative correlation between astrocytic C3 expression and RGC density, demonstrating the relationship between enhanced optic nerve astrocyte complement expression and RGC loss. Interestingly, this sex difference was a unique feature of astrocytes of ON and was not observed in spinal cord.

## Discussion

The current study extends our previous findings defining astrocyte-specific transcriptomes across multiple CNS regions in the EAE mouse model of MS^1^, here to the optic nerve. Distinct from other regions, the Complement Cascade Pathway was the most upregulated pathway in EAE optic nerve astrocytes (Fig. 3).

The role of complement in health and disease has been widely investigated^32,33^. Complement activation is crucial for the pathogenicity of NMO, an autoimmune disease predominantly affecting optic nerve and spinal cord^34^. Deposition of complement was also described in EAE and in chronic MS plaques, where it was proposed to mediate plaque expansion and progression^35,36^. However, mechanisms and cellular interactions through which complement may drive progression remains unclear. Astrocytes are known to play both beneficial and detrimental roles in disease^13–15,37^. Despite evidence of a neuroprotective role of astrocytic C3 expression early in disease^38^, long term aberrant C3 expression may result in autoimmunity, defective clearance of myelin debri, and synaptic stripping^16,21,35,39^. Here, we found that among the complement cascade genes, *C3* was the most significantly upregulated gene in optic nerve astrocytes in late EAE. C3 expression in astrocytes has been described in both brain aging and chronic neurodegenerative diseases^16,40,41^. Translating back to MS, we speculate that expression of C3 in astrocytes could play a role in MS disease progression in older MS patients.

In parallel with C3 overexpression in EAE optic nerve astrocytes, we also found overexpression of its receptor C3aR in microglia/macrophages. A recent study reported microglia-astrocyte cross-talk through complement activation in an Alzheimer’s disease mouse model, where aberrant expression of astrocytic C3 impaired microglia Aβ phagocytosis in a C3aR dependent manner^21^. In demyelinating disorders, defective clearance of myelin debri by microglia has been associated with impaired remyelination^42,43^. Whether a similar complement-dependent intercellular cross talk (C3-C3aR) may be involved in defective debri clearance and inefficient remyelination in optic neuritis warrants further investigation.

Our RNA-seq analysis also revealed upregulation of *Thbs1* in optic nerve astrocytes in EAE. We found astrocytic expression of THBS1 increased early (EAE day 21) and decreased late (EAE day 50), consistent with the hypothesis of a more beneficial role of optic nerve astrocytes early in EAE. Given the role of astrocytic C3 and THBS1 in RGC synaptic stripping and plasticity, respectively^32,44^, we extended the investigation of C3 and THBS1 to the retina. Retina presents an organized laminar structure of synaptic connections among RGCs, interneurons and photoreceptors. It lacks myelin and is remote from the inflammatory lesions in optic nerve during EAE. Nevertheless, neuronal and axonal loss occurs in the retina. Retinal astrocytes form a thin layer confined to the RNFL. We found no increase in expression of C3 in astrocytes during EAE in retina at any timepoint. Activated microglia and classical inflammatory mediators (IL1α, TNFα, C1q) are thought to upregulate astrocytic C3 expression^16^. A small but significant increase in microglia activation in the retina was reported early in EAE^45^, but not late^46^. Thus, our observation of no increase in C3 in retina late in EAE is consistent with the lack of microglia activation and immune cell infiltration in the retina. Conversely, synaptogenic THBS1 was highly expressed early (EAE day 21) and decreased late (EAE day 50) in retina. This suggests that, unlike astrocytic C3 induction, astrocytic THBS1 expression is independent of inflammation. The neuroprotective synaptogenic potential of THBS1 is relevant in retina, a region rich with synapses. Conversely, the role of THBS1 in optic nerve is less apparent. Previous studies reported the presence of synaptic activity in CNS white matter oligondendrocyte precursors cells (OPCs) and showed that synaptic activity between OPCs and demyelinating axons regulated remyelination^47,48^. Whether THBS1 plays a role in promoting synapses between demyelinated axons and OPCs to modulate remyelination in optic nerve during EAE is possible.

Evidence here that the astrocyte reactivity state differs between optic nerve and retina, is consistent with astrocyte heterogeneity across CNS regions^1,12,49^. Such differences may be due to the difference in distribution of mylein and inflammation, or to different types of astrocytes populating each compartment (protoplasmatic in retina and fibrous in optic nerve), or both. Astrocyte heterogeneity exists not only across CNS regions, but also between sexes^50,51^. Here, we found that in EAE optic nerve, females showed more of an increase in C3 expressing astrocytes, while males showed more increase in THBS1 expressing astrocytes. This observation was accompanied by worse RGC body and axonal loss in females compared to males, with a significant negative correlation between astrocytic C3 expression and RGC density, suggesting a connection between enhanced optic nerve astrocyte complement expression and RGC loss^16,52^.

Females are known to be more susceptible than males to MS^53,54^ and within MS patients optic neuritis affects more women than men. Also, there is a 7:1 ratio of women to men for NMO^28,29^. Our findings, combining RNA sequencing analysis of ON astrocytes in females, followed by investigation of lead targets by immunohistochemistry in ON and retina of both females and males, reveal an interesting difference in astrocyte reactivity in female versus male optic nerve. This sexual dimorphism may be due to differences in sex hormones or sex chromosomes^54,55^, either of which could modulate astrocyte reactivity in optic nerve in EAE. The fact that we did not observe this sex difference in spinal cord, makes the optic nerve unique and further underscores the heterogeneity of astrocytes across CNS regions^1,49^. Regardless of the etiology of the sex difference, the evidence that C3 expressing astrocytes in optic nerve are found more in females than in males suggests that a treatment targeting expression of C3 in astrocytes during optic neuritis may be more effective in females than in males. Together, this underscores the importance of precision medicine using a disability-specific and sex-specific approach.

## Material and Methods

### Animals

All mice used in this study were young adult females and males (8–12 week old) on a C57BL/6 background. RiboTag mice (B6N.129-*Rpl22tm1*.*1Psam*/J) were purchased from Jackson Laboratory and back crossed with mGFAP-Cre mice line 73.12, kindly donated by Prof. Michael Sofroniew (UCLA). All mice were housed in a 12 h dark/light cycle in a specific-free-pathogen facility with free access to food and water. All experiments were conducted according to protocols approved by the Animal Research Committee of the Office for Protection of Research Subjects at University of California Los Angeles.

### EAE induction and clinical scoring

Active EAE was induced with myelin oligodendrocyte glycoprotein (MOG), amino acids 35 to 55, in wild type and GFAP-Cre (73.12) RiboTag mice, as previously described^1^. Briefly, young (8–12 week old) mice were immunized on days 0 and 7 with subcutaneous injections of MOG (200 μg/animal, American Peptides) emulsified in complete Freund’s adjuvant (CFA), supplemented with Mycobacterium tuberculosis H37Ra (200 μg/ animal; Difco Laboratories). Pertussis toxin (500 ng/mouse, List Biological Laboratories, Inc., Campbell, CA) was injected intraperitoneally (i.p.) on days 0 and 2. Animals were monitored daily for EAE signs as described^56^.

### Spectral Domain Optical Coherence Tomography (SD-OCT)

Longitudinal SD-OCT examination of the retinal nerve fiber layer (RNFL) was performed at days 8, 21 and 50 post EAE immunization using Envisu SD-OCT device from Bioptigen, available at the UCLA Live Imaging and Functional Evaluation Core at the Jules Stein Eye Institute, as described^57^. At each time point, EAE and healthy mice (n = 5 per group) were anaesthetized by i.p. application of ketamine (100 mg/kg, VETone) and xylazine (10 mg/kg, Lloyd Lab). Pupils were dilated by topical administration of 1% tropicamide (Akorn Inc.). RNFL thickness was determined by performing radial scans of the retina (1.8 mm diameter, ON head centered, 1000 A scans x 45 B scans x 24 frames). RNFL thickness was manually measured using ImageJ. The optic disc center was identified, and mean values for RNFL layer thickness were obtained along a distance of 0.05 mm from the optic disc center, as described^58^.

### Histology and immunohistochemistry

Mice were sacrificed by lethal dose of isoflurane and perfused transcardially with ice cold 1X PBS, followed by 10% formalin (Sigma-Aldrich). For RGC axonal count, ONs were processed as previously described^59^. Briefly, ONs were removed and post-fixed in ice-cold 5% glutaraldehyde in 0.1 M phosphate buffer (PBS) for 48 hours (h) at 4 °C, immersed in 1% osmium tetroxide (pH 7.4) for 3 hours, dehydrated in ascending grades of ethanol (75%, 95% and 100%) and finally embedded in epoxy resin. Semi-thin (1 um) ON coronal sections were collected at the ultramicrotome (Leica), dried onto slides and stained with 1% Toluidine Blue (Sigma-Aldrich). Imaging was performed on 3 to 5 cryosections (2 to 3 mm distal from the eye globe) per biological sample. Images were captured at 63x magnification using bright field microscopy. Axonal survival was assessed by automated axonal count using Image J and graphed as axon density, which is the number of axons per µm^2^, as previously described^59^. For RGC cell body count, eyes were enucleated and, following posterior eye cup dissection and lens removal, retinas were carefully dissociated from the underlying retinal pigment epithelium, flat mounted onto nitrocellulose membrane discs (Millipore) and post-fixed overnight in cold 10% formalin at 4 °C, as described^60^. To obtain retinal, ON coronal sections and spinal cord transverse sections, posterior eyecups,ON and spinal cord, respectively, were post-fixed in formalin 10% overnight at 4 °C, cryoprotected in 30% sucrose overnight at 4 °C and embedded in optimal cutting temperature compound (Sakura Tissue Tek). Posterior eyecups, ONs and spinal cords were cryosectioned at 14 um, 10 um and 40 um, respectively.

For immunohistological staining, the following primary antibodies were used: mouse anti-BIII tubulin (Promega, 1:1000); goat anti-Brn3a (SantaCruz Biotech, 1:100); rabbit anti-RBPMS (Santa Cruz Biotech, 1 :500); rat anti-CD45 (BD Pharmigen, 1:500); mouse anti-PLP (Millipore, 1:50); rat anti-GFAP (ThermoFisher, 1:500); mouse anti-GFAP (Abcam, 1:500); goat anti-LCN2 (R&D system, 1:50); mouse anti-HA (HA.11 Clone 16B12, Biolegend, 1:500); rabbit anti-GSTπ (Enzo Life Science, 1:500); rabbit anti-NF200 (Sigma-Aldrich, 1:500); rabbit anti-Iba1 (Wako, 1:400); rat anti-C3aR (Hycult Biotech, 1:100); rat anti-MHCII (BioLegend, 1:400); rat anti-CD3 (BD Pharmigen, 1:2000); rabbit anti-RLBP1 (Proteintech, 1:50), rabbit anti-C3 (Hycult Biotech, 1:50); mouse anti-THBS1 (Santa Cruz Biotech, 1:50). Nuclei were counterstained with DAPI. Imaging was performed on 3 to 5 cryosections per biological sample. Specifically, 3 areas per ON cryosection (proximal to the ONH, central and proximal to the ON chiasm), 2 areas per retina cryosection (on the left and right side of the ONH) and one area per spinal cord cryosection (ventral side) were imaged. Images were captured at 10x magnification using Olympus BX51 fluorescence microscope with a DP50 digital camera and processed using ImageJ. Positive labelling in a specific cell type (i.e C3 expression in GFAP^+^ astrocytes) was quantified and graphed as area fraction, which is the percentage of double positive labelling in the cell type of interest within the imaged area (% C3^+^GFAP^+^/GFAP^+^), as previously described^1,61^.

### Co-immunoprecipitation and astrocyte enriched RNA isolation

Female EAE and age- matched normal control GFAP-Cre RiboTag mice were sacrificed at EAE day 50 post immunization. After terminal anesthesia with overdose of isoflurane, mice were perfused with ice-cold 1X PBS, followed by ice-cold 1% paraformaldehyde/1X PBS. ONs were rapidly removed and snap frozen in liquid nitrogen and stored at −80 °C till further processing. In order to obtain sufficient RNA, ONs from 2 mice were pooled for each sample. Normal control GFAP-Cre RiboTag mice were used to confirm specificity of HA targeting to astrocytes in ON. Tissue homogenization and HA immunoprecipitation (HA-IP) of astrocyte enriched ribosomal RNA were performed as previously described^1^. Briefly, frozen ONs were homogenized in 1 ml buffer (50 mM Tris-HCl, 100 mM KCl, 12 mM MgCl2, 1% NP40, 1 mM DTT, 1x Proteinase Inhibitors, 200 U/ml RNAsin, 100 ug/ml cyclohexamide and 1 mg/ml heparin). The homogenate was centrifuged at max speed for 10 minutes at 4 °C, the supernatant collected and incubated overnight at 4 °C with pre-washed anti-HA conjugated to protein A/G magnetic beads (Pierce). The following day, the non-precipitated flow-through (FT) from each HA-IP sample was collected and RNA was purified for enrichment analysis using RNeasy MicroKit (Qiagen), according to manufacturer’s instructions. Magnetic beads were washed 3 times in high salt buffer (50 mM Tris-HCl, 300 mM KCl, 12 mM MgCl2, 1% NP40, 1 mM DTT and 100 ug/ml cyclohexamide). Beads were then incubated with 25 ul Proteinase K (4 mg/ml, Zymo Research) at 55 °C for 30 minutes, to increase the RNA recovery yield. After incubation, 300 ul of TriReagent (Zymo Research) were added to the beads, which were then thoroughly vortexed and pelleted using a magnetic separator. The supernatant was transferred into a new tube and HA-IP RNA was purified using Direct-zol RNA microPrep (Zymo Research), according to manufacturer’s instructions. RNA integrity of HA-IP samples was assessed by Agilent 2100 Bioanalyzer using RNA pico Chip.

### Library construction and high throughput RNA sequencing

The RNA sequencing library was made using Nugen Oviation 2 RNA-Seq System V2 kit (Nugen) followed by KAPA Stranded RNA-Seq Kit (Kapa Biosystems). Barcoded adaptors were used for multiplexing samples in one lane. Sequencing was performed on Illumina HiSeq3000 for a single end 1 × 50 run. Data quality check was done on Illumina SAV. De-multiplexing was performed with Illumina Bcl2fastq2 v 2.17 program. These procedures were performed in the UCLA Technology Center for Genomics and Bioinformatics.

### High throughput RNA sequencing analysis

Statistical analyses and production of figures were performed in R (R Core Team, 2018, http://www.R-project.org/). Qualities of raw sequence data were examined using FastQC (http://www.bioinformatics.babraham.ac.uk/projects/fastqc/), and Trimmomatic was used for cleaning. R package “QuasR” was used for the read alignment to the mouse genome (mm10) followed by the counting at the gene level. We assumed read counts followed a negative binomial distribution and constructed generalized linear models based on this negative binomial distribution assumption. Differentially expressed genes between normal and EAE mice were identified using R package “edgeR.” False discovery rate (FDR) of 0.1 was used as a threshold of differentially expressed genes. Canonical pathway enrichment analysis was performed for differentially expressed genes (FDR <0.1, logFC >1 or logFC <−1) in each tissue using Ingenuity Pathway Analysis (QIAGEN, Redwood City, www.qiagen.com/ingenuity) as described^1^.

### Quantitative RT-PCR

Enrichment and de-enrichment of cell specific gene transcripts in HA-IP RNA relative to FT RNA, as well as validation of RNA sequencing data were confirmed by quantitative RT-PCR (qPCR) using Power Syber Green RNA to ct-1 step kit and TaqMan RNA to ct-1 step kit (Applied Byosystem), according to manufacturer’s instructions. The following primers and Taqman probes were used:

*Lcn2* (Thermo Fisher, Mm01324470)

*C3* (Thermo Fisher, Mm01232779_m1)

*SerpinG1* (Thermo Fisher, Mm00437835_m1)

*Actβ* (Thermo Fisher, Mm04394036_g1)

*Thbs1*(fwd: GAGCAGGAGGTCCACTCAGA; rev: CCACAGTTCCTGATGGTGAA)

*Actβ* (fwd: GGTCCTAGCACCATGAAGA; rev: ACTCCTGCTTGCTGATCCAC)

The efficiency of each set of primers was assessed by qPCR on a serial dilution of mouse ON total cDNA and was confirmed to be above 90%. All gene expression levels were normalized to levels of *β*-*Actin* and expressed as fold change relative to control by using ΔΔCt method for Taqman assay and standard curve method for Syber Green assay.

### Statistical analysis

A minimum of 3–5 animals per group were used for all experiments. All data are presented as mean ± s.e.m. Pairwise comparisons were analyzed using a two-tailed unpaired Student’s *t* test, whereas multiple comparisons were analyzed using two-way and one-way ANOVA. Dunnett’s was used as post hoc test for one-way ANOVA. Sidak’s or Tukey’s were used as post hoc test for two-way ANOVA. Linear regression analysis was performed to determine correlation between C3 expression in astrocytes and RGC loss. Correlation was examined using Pearson’s correlation coefficient. Statistical analysis was performed with GraphPad Prism 6 (version 6.1) using confidence interval of 0.05. *P* values higher than or equal to 0.05 were considered not significant.

## Supplementary information

SI Tassoni A et al

## Data Availability

Female optic nerve RNA-Seq dataset generated during this study was published in our previous paper^1^ and is available in the GEO (Gene Expression Omnibus; www.ncbi.nlm.nih.gov/geo/) under the accession number GSE100294.
