# Supplementary material for: The astrocyte transcriptome in EAE optic neuritis shows complement activation and reveals a sex difference in astrocytic C3 expression
Source: Sci Rep. 2019 Jul 10;9:10010. doi: 10.1038/s41598-019-46232-6 (PMC6620300; doi:10.1038/s41598-019-46232-6)
Supplement: Supplementary file 1 — SI Tassoni A et al [file 41598_2019_46232_MOESM1_ESM.pdf]

Supplementary Information

The astrocyte transcriptome in EAE optic neuritis shows complement activation and reveals sex difference in astrocytic C3 expression

Alessia Tassoni[1], Vista Farkhondeh[1],Yuichiro Itoh[1], Noriko Itoh[1], Michael V. Sofroniew[2], Rhonda R. Voskuhl[1*]

Supplemental Fig.1

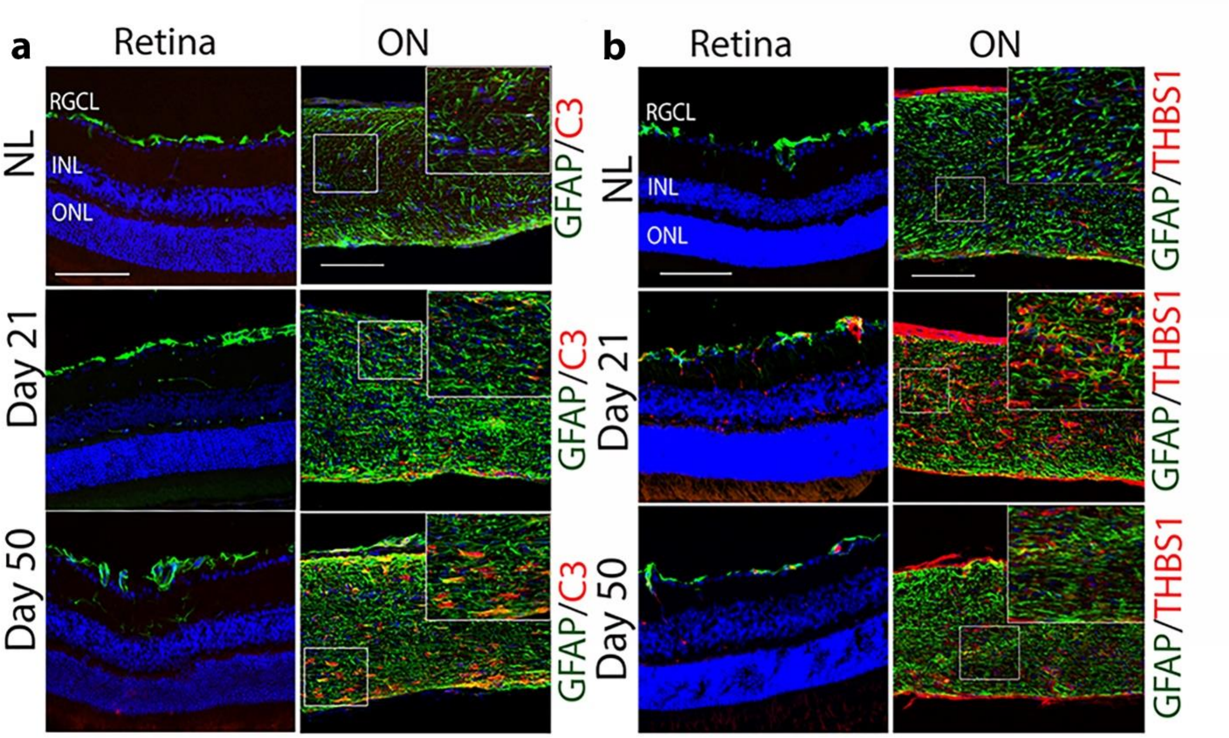

Supplemental Figure 1. Regional difference in the anterior visual pathway in EAE optic neuritis. ***a,*** Immunofluorescence showing expression level of C3 (red) in retina and ON astrocytes (GFAP, green) at EAE day 21 and 50, compared to NL. No increase in C3 expression was detected in retina astrocytes. ***b,*** Immunofluorescence showing expression level of THBS1 (red) in retina and ON astrocytes (GFAP, green) at EAE day 21 and 50, compared to NL. Nuclei were counterstained with DAPI (blue). Scale bar 100 μm

Supplemental Fig.2

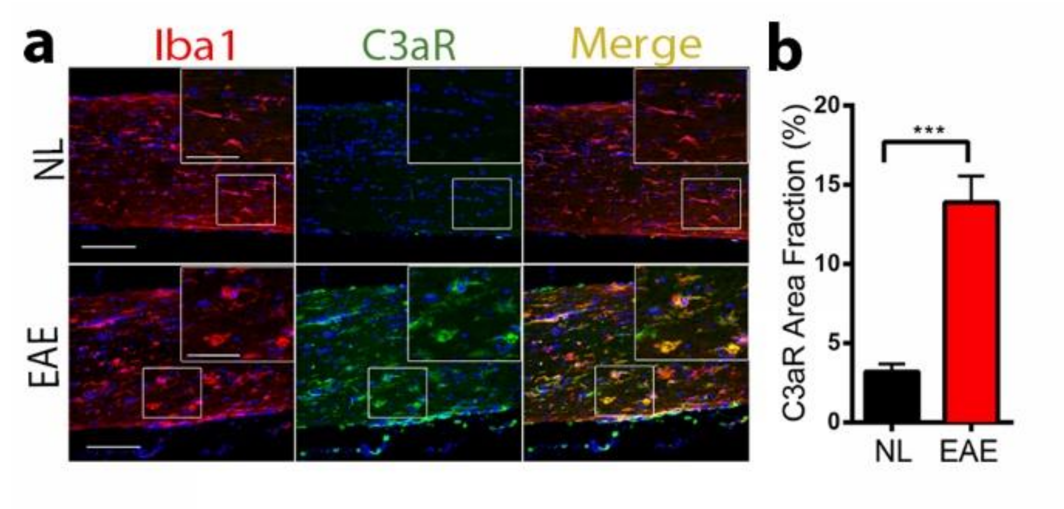

Supplemental Figure 2. Upregulation of C3aR in ON microglia/macrophages. *a*, Immunofluorescence showing expression level of C3aR (green) in ON microglia/macrophage (Iba1, red) in EAE and NL. Nuclei were counterstained with DAPI (blue). Scale bar 100 μm. ***b*,** Quantitative analysis showing significant upregulation of C3aR expression in ON in EAE (red bar) compared to NL (black bar). Data represent mean ± s.e.m. (n=5 per group), Student's t-test, ***p<0.001

Supplemental Fig. 3

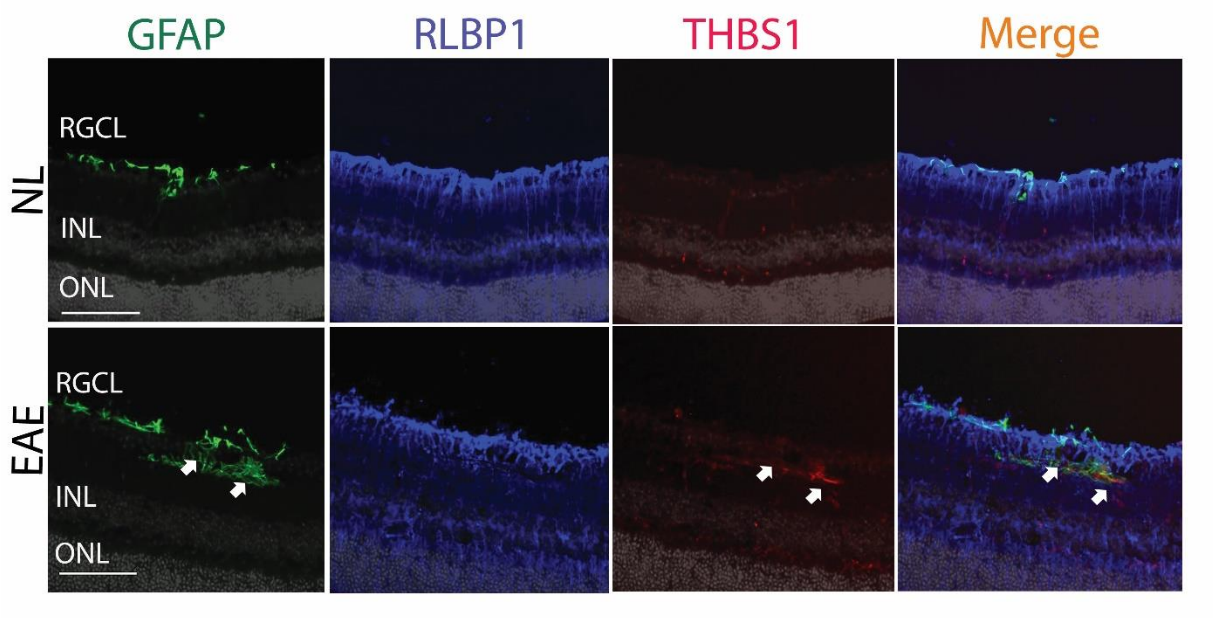

Supplemental Figure 3: THBS1 expression in retinal astrocytes in EAE optic neuritis. Immunofluorescence investigating expression level of THBS1 (red) in retina astrocytes (GFAP, green) and Muller cells (RLBP1, blue) in EAE, compared to NL. Increased THBS1 labelling was observed in retina astrocytes (arrows) during EAE. Nuclei were counterstained with DAPI (gray). Scale bar 100 μm.

Supplemental Fig. 4

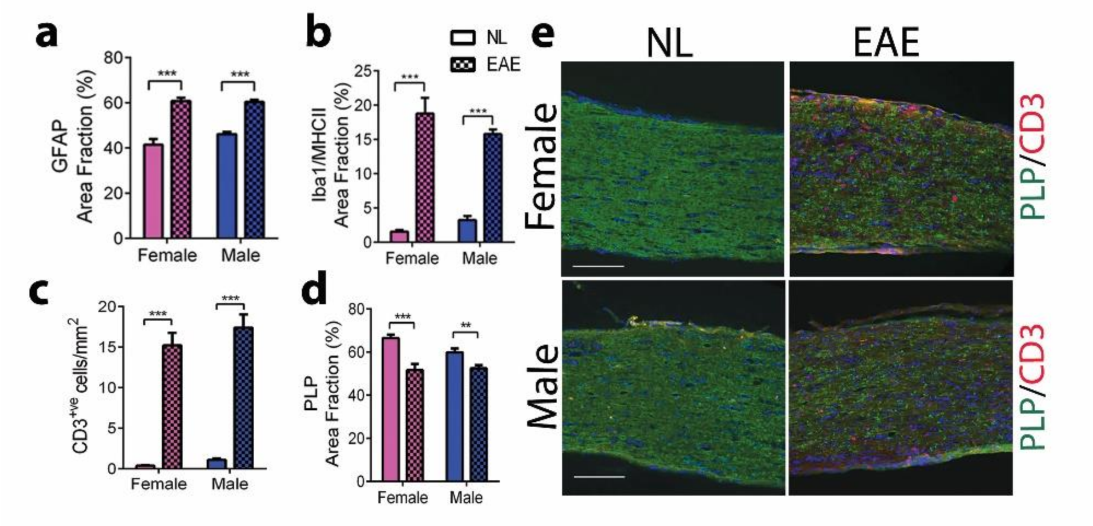

Supplemental Figure 4: Sex difference in ON inflammation and demyelination in EAE optic neuritis. Immunofluorescence quantitative analysis showing no sex differences in reactive astrocytes (***a***), microglia/macrophage reactivity (***b***), T cell infiltration (***c***) and demyelination (***d***) in females (pink bars) and males (blue bars) during EAE (checked bars) compared to NL (full bars). ***e***, Representative images showing myelin integrity (PLP, green) and T cell infiltration (CD3, red) in ON in females and males in EAE and NL. Nuclei were counterstained with DAPI (blue). Scale bar 100 μm. For all bar graphs, data represent mean ± s.e.m. (n=5-7 per group), Multiple t test **p<0.01, ***p<0,0001, followed by Two Way ANOVA with Tukey's multiple comparison test (Interaction, *non significant*).

Supplemental Fig. 5

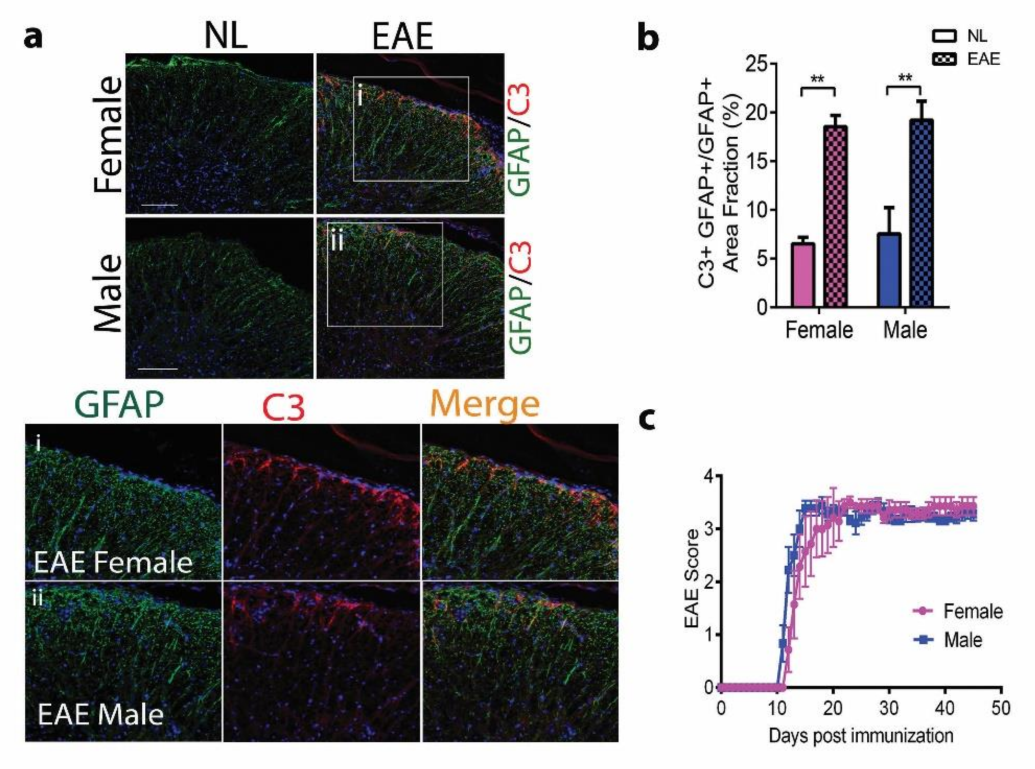

Supplemental Figure 5: Sex difference in astrocytic C3 expression in spinal cord during EAE. ***a***, Representative images showing expression of C3 (red) in spinal cord astrocytes (GFAP, green) in females (i) and males (ii) in EAE and NL. Nuclei were counterstained with DAPI (blue). Scale bar 100 µm. ***b***, Quantitative analysis showing no sex differences in expression of C3 expressing in spinal cord astrocytes in females (pink bars) and males (blue bars) during EAE (checked bars) compared to NL (full bars). ***c*,** EAE clinical score in females (pink) and males (blue). Data represent mean ± s.e.m. (n=5-7 per group), Multiple t test **p<0.01, ***p<0,0001, followed by Two Way ANOVA with Tukey’s multiple comparison test (Interaction, *non significant*).
